# Association Analysis of Methylenetetrahydrofolate Reductase Common Gene Polymorphisms with Breast Cancer Risk in an Iranian Population: A Case-Control Study and a Stratified Analysis

**DOI:** 10.31557/APJCP.2020.21.9.2709

**Published:** 2020-09

**Authors:** Mohammad Karimian, Nasrin Rezazadeh, Tahereh Khamehchian

**Affiliations:** 1 *Department of Molecular and Cell Biology, Faculty of Basic Sciences, University of Mazandaran, Babolsar, Iran. *; 2 *Department of Pathology, School of Medicine, Kashan University of Medical Sciences, Kashan, Iran. *

**Keywords:** Breast cancer, MTHFR, genetic polymorphism, risk factor

## Abstract

Genetic polymorphisms in the methylenetetrahydrofolate reductase (MTHFR) gene may alter the risk of breast cancer. This study aimed to investigate the association of MTHFR C677T and A1298C genetic polymorphisms with breast cancer risk in case-control studies which was followed by stratified analysis. In the case-control study, 300 subjects including 150 women with breast cancer and 150 healthy women were enrolled. After blood sample collection, the C677T and A1298C polymorphisms genotyping were done by the PCR-RFLP method. Our data revealed a significant association between MTHFR C677T and A1298C polymorphisms and breast cancer risk. But, as a preliminary study, stratified analysis revealed no significant association between C677T and A1298C polymorphisms and tumor size and also lymph node metastasis in breast cancer. According to the mentioned findings, the C677T and A1298C polymorphisms in the MTHFR gene could be molecular risk factors for breast cancer in our studied population. However, further studies with larger sample sizes are required to obtain a more accurate conclusion in stratified analysis.

## Introduction

Breast cancer is one of the most common causes of death in women worldwide (Bray et al., 2018). This malignancy is also considered as the most prevalent cancer in women worldwide (Fares et al., 2019). Breast cancer is a complex and multifactorial disease that could be affected by various genetic and environmental factors. For instance, age over 40 years, history of some proliferative breast diseases, history of breast cancer in the first-degree family, early menstruation, late menopause, nulliparity, and higher socioeconomic status (Kamińska et al., 2015). Genetic factors also play an important role in tumor formation and progression. For example, aneusomy of chromosome 17, deletion of loci at 16q, 11q, 6q, and 3p and polymorphisms in genes involved in the key cellular pathways can be the main risk factors for breast cancer (Newsham, 1998). One of the important cellular pathways is the folate metabolism cycle which includes many genes such as methylenetetrahydrofolate reductase (MTHFR), methionine synthase (MTR), and methionine synthase reductase (MTRR). The role of the MTHFR enzyme is to convert 5,10-methylenetetrahydrofolate (5,10-MTHF) to 5-methyltetrahydrofolate (5-MTHF), which is a common substrate for methylation of homocysteine to methionine. The methyl group of methionine is used to form S-adenosyl methionine which is a methyl donor for DNA and protein methylation (Shrubsole et al., 2004). This gene consists of numerous single nucleotide polymorphisms (SNPs). The C677T (rs1801133) and A1298C (rs1801131) genetic variations are the most common polymorphisms of the MTHFR gene which may contribute to breast cancer risk. The C677T polymorphism results in alanine to valine replacement at the codon 222. While the A1298C polymorphism replaces glutamate to alanine at codon 429 (Hesari et al., 2019).

There are some studies that investigated the correlation of two common polymorphisms (C677T and A1298C) in MTHFR gene with the breast cancer risk. Kaya et al in 2016 reported no significant association between C677T polymorphism and breast cancer risk (Kaya et al., 2016). Also, Papandreou et al. in 2012 reported no significant association between A1298C and breast cancer risk (Papandreou et al., 2012). However, Ergul et al., (2003) reported a significant association between two mentioned SNPs and breast cancer risk (Ergul et al., 2003). Given the above contradictory results, the purpose of this study is to determine the association of C677T and A1298C SNPs of the MTHFR gene with breast cancer risk in a case-control study which is followed by stratified analysis.

## Materials and Methods

*Subjects*

This study consisted of 150 breast cancer patients and 150 cancer-free and age-matched women. Case individuals were the patients referring to Hospitals in Isfahan province and their breast cancer was confirmed by pathological tests. They completed a questionnaire containing information about their BMI, history of drug, age, and family history of breast cancer. Patients with diseases such as diabetes, other cancers, CHD, and etc. were excluded from the study. The control group was composed of healthy women without a family history of cancer and other disorders who were referring to the same hospitals for routine examinations. Both cases and controls were belonging to a local area with the same ethnicity. All participants signed an informed consent form and this project was approved by the ethics committee of the Kashan University of Medical Sciences (IR.KAUMS.MEDNT.REC.1397.39). Approximately 2 ml of blood was taken from each woman and transferred to an EDTA-coagulated tube and stored at -20°C until DNA extraction. 

*DNA extraction and SNPs genotyping*

DNA was extracted from peripheral blood samples by a commercial DNA extraction kit (Bioneer, Daejeon, Korea). polymerase chain reaction-restriction fragment length polymorphism (PCR-RFLP) method was used to C677T and A1298C SNPs genotyping. For this purpose, at first, forward and reverse oligonucleotide primers were deduced from our previous study (Karimian and Colagar, 2016) and were ordered from Bioneer Company to amplify fragments containing two mentioned SNPs. Approximately 5 microliters of twice-distilled water were added to 0.2 ml micro-tubes to reach a final volume of 20 µl. Then, 10µl of 2X premix PCR mixture, 0.3 µM of each pair of primers, and 60ng of DNA template were added to the micro-tubes. After a short vortex and then spin the samples, PCR was performed in a Peqlab thermal cycler (PEQLAB Biotechnologie GmbH, Germany). All PCR reagents were ordered from Cinnagen company (Cinnagen, Tehran, Iran). The thermal cycling conditions for PCR were as follows: initial denaturation at 94°C for 4 min, then 35 cycles of denaturation at 94°C for 30 min, annealing at 63°C for 30 min for C677T polymorphism and 67°C for A1298C, and elongation at 72°C for 30 min, with a final extension at 72°C for 5 min. The PCR products were digested by the HinfI enzyme for the C677T variant and MboII enzyme for A1298C SNP according to manufacturer’s instruction (Fermentas, Waltham, Massachusetts, United States).

**Figure 1:**
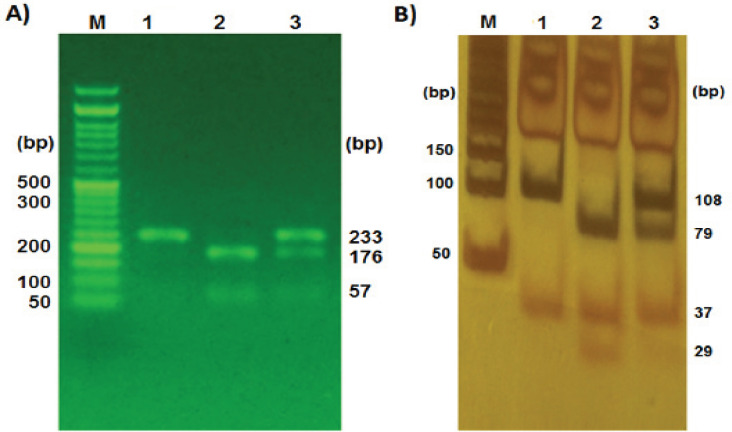
Polymorphism Analysis of *MTHFR*-C677T and *MTHFR*-A1298C. A) The PCR products containing C677T variation which digested with Hinf I restriction enzyme (lane M, DNA marker; Lane 1, CC genotype; Lane 2, TT genotype; Lane 3, CT genotype); B) The PCR products containing A1298C variation which digested with MboII restriction enzyme (lane M, DNA marker; Lane 1, CC genotype; Lane 2, AA genotype; Lane 3, AC genotype)

PCR products were electrophoresed on 1% agarose gel and the fragments containing C677T and A1298C variations produced the bands with a length of 233-bp and 143-bp, respectively. The genotypes of C677T polymorphism was detected by 2% agarose gel electrophoresis. The fragment with TT genotype was digested into two 176-bp and 57-bp fragments, whereas in CT genotype was digested into 233-bp, 176-bp, and 57-bp fragments. In CC genotype, only one band containing the 233-bp fragment was observed because there was no restriction site for HinfI (Figure 1). Regarding A1298C polymorphism, the 143-bp digested fragment on 8% polyacrylamide gel demonstrated two bands of 108-bp and 37-bp for CC genotype, three bands of 37-bp and 79-bp, and 29-bp for AA genotype, and four bands of 108-bp, 79-bp, 37-bp, and 29-bp for AC heterozygote genotype (Figure 1). For PCR-RFLP verification, three samples of PCR products with CC, CT, and TT genotypes for C677T polymorphism and three samples with CC, AC, and AA genotypes for A1298C polymorphism were respectively sequenced using forward and reverse primers by Bioneer Company.

*Statistical analysis*

Statistical tests were performed using SPSS 22 software (SPSS Inc., IBM Corp Armonk, NY, USA). The Chi-square test was used to compare alleles and genotypes frequencies in case and control groups. This test was also used to evaluate Hardy-Weinberg equilibrium in both the cases and healthy control groups. A binary logistic regression was used to obtain odds ratio (OR) and 95% confidence interval (CI) for investigation of the association between the two polymorphisms and the risk of breast cancer in overall and stratified analyses. A two-tailed p-value of less than 0.05 was considered statistically significant.

## Results

*Association of C677T and A1298C SNPs with breast cancer*

The results of genetic association analysis for C677T and A1298C polymorphisms are summarized in Table 1. Distribution of genotypes in case and control groups for both mentioned SNPs was consistent with Hardy-Weinberg equilibrium criteria. Concerning C677T polymorphism, the C allele was more prevalent than another allele. The frequency of allele T in the patient group was significantly higher than the control group and statistical analysis showed that there was a significant correlation between T allele and increased risk of breast cancer (OR= 2.31, 95%CI= 1.58-3.38, p< 0.0001). The frequency of CT genotype in control and patient groups was 25.33% and 47.33%, respectively while these ratios for the TT genotype were 5.40% and 10.00%, respectively. Statistical analysis showed a significant association between CT (OR= 3.04, 95%CI= 1.84-5.02, p< 0.0001) and TT (OR= 3.05, 95%CI= 1.22-7.59, p= 0.017) genotypes and increased risk of breast cancer. Besides, carriers of T allele were at high risk for breast cancer susceptibility (OR= 2.31, 95%CI= 1.58-3.38, p< 0.0001). Regarding A1298C polymorphism, our data showed that the C allele was more prevalent than A allele in the case and control groups. The frequency of genotype AC in control and case group was 38.67% and 39.33%, respectively while these ratios for CC genotype were 8.66% and 16.67%, respectively. Statistical analysis showed that the CC genotype has a significant correlation with increased risk of breast cancer (OR= 2.30, 95%CI= 1.09-4.85, p= 0.028). Also, the frequency of the C allele in the patient group was significantly higher than the control group and subsequent analysis showed a significant association between C allele and increased risk of breast cancer (OR= 1.47, 95%CI= 1.04-2.07, p= 0.029).

*Stratified analysis*

The genotypes distribution for C677T and A1298C polymorphisms were evaluated for size of tumor and metastasis of lymph node in breast cancer subjects (Table 2). Concerning C677T polymorphism, we found no true associations between this variation and tumor size of breast malignancy (CT vs. CC: OR= 1.31, 95%CI= 0.63-2.72, p= 0.477; TT vs. CC: OR= 1.70, 95%CI= 0.53-5.48, p= 0.371; CT+TT vs. CC: OR= 1.37, 95%CI= 0.68-2.76, p= 0.381). Such results were observed for lymph node metastasis (CT vs. CC: OR= 1.12, 95%CI= 0.57-2.23, p= 0.733; TT vs. CC: OR= 1.56, 95%CI= 0.48-5.07, p= 0.464; CT+TT vs. CC: OR= 1.19, 95%CI= 0.62-2.29, p= 0.604). Also, we did not observe any significant correlation between A1298C variation and tumor size and also lymph node metastasis of breast malignancy (Table 2). Our data showed that this variation could not be associated with the tumor size in three genetic model analysis. The same results were observed for lymph node metastasis in three genetic models analysis (AC vs. AA: OR= 1.02, 95%CI= 0.50-2.09, p= 0.963; CC vs. AA: OR= 0.60, 95%CI= 0.24-1.52, p= 0.280; AC+CC vs. AA: OR= 0.87, 95%CI= 0.45-1.67, p= 0.670).

**Table 1:** Association of *MTHFR*-C677T and *MTHFR*-A1298C polymorphisms with breast cancer risk

Genotype/Allele	Case (n=150)	Control (n=150)	χ2 value	OR (95% CI)	*P*-value
A) *MTHFR*-C677T					
CC	64 (42.67%)	104 (69.33%)	-	-	-
CT	71 (47.33%)	38 (25.33%)	19.35	3.04 (1.84-5.02)	< 0.0001*
TT	15 (10.00%)	8 (5.40%)	6.14	3.05 (1.22-7.59)	0.017*
CT+TT	86 (57.33%)	46 (30.66%)	21.65	3.04 (1.89-4.88)	< 0.0001*
C	199 (66.33%)	246 (82.00%)	-	-	-
T	101 (33.67%)	54 (18.00%)	19.22	2.31 (1.58-3.38)	< 0.0001*
B) *MTHFR*-A1298C					
AA	66 (44.00%)	79 (52.67%)	-	-	-
AC	59 (39.33%)	58 (38.67%)	0.63	1.22 (0.75-1.98)	0.429
CC	25 (16.67%)	13 (08.66%)	4.95	2.30 (1.09-4.85)	0.028*
AC+CC	84 (56.00%)	71 (47.34%)	2.26	1.42 (0.90-2.23)	0.134
A	191 (63.67%)	216 (72.00%)	-	-	-
C	109 (36.33%)	84 (28.00%)	4.77	1.47 (1.04-2.07)	0.029*

OR, odds ratio; CI, confidence interval. *Significant differences between cases and controls.

**Table 2:** The Association Analysis between *MTHFR*-C677T and *MTHFR*-A1298C Variation with Two Cclinical Characteristics of Breast Cancer

A) *MTHFR*-C677T				
Characteristics	Genotype distributions			
	CC	CT	TT	CT+TT
Tumor size (cm)				
≥2/<2	18/46	24/47	6/9	30/56
OR (95% CI)	1.0 (reference)	1.31 (0.63-2.72)	1.70 (0.53-5.48)	1.37 (0.68-2.76)
p-value	-	0.477	0.371	0.381
Lymph node metastasis				
Yes/No	36/28	42/29	5-Oct	52/34
OR (95% CI)	1.0 (reference)	1.12 (0.57-2.23)	1.56 (0.48-5.07)	1.19 (0.62-2.29)
p-value	-	0.733	0.464	0.604
B) *MTHFR*-A1298C				
Characteristics	Genotype distributions			
	AA	AC	CC	AC+CC
Tumor size (cm)				
≥2/<2	16/50	21/38	14-Nov	32/52
OR (95% CI)	1.0 (reference)	1.73 (0.80-3.75)	2.46 (0.93-6.48)	1.92 (0.94-3.93)
p-value	-	0.167	0.07	0.073
Lymph node metastasis				
Yes/No	40/26	36/23	13-Dec	48/36
OR (95% CI)	1.0 (reference)	1.02 (0.50-2.09)	0.60 (0.24-1.52)	0.87 (0.45-1.67)
p-value	-	0.963	0.28	0.67

Cm, centimeter; OR, odds ratio; CI, confidence interval

## Discussion

In the present study, we evaluated the association of two common variations in the MTHFR gene with the risk of breast cancer in a case-control study which was followed by stratified analysis. Our case-control study revealed a significant association between A1298C and C677T genetic variation and increased risk of breast cancer in our studied population. But, there are some similar studies with inconsistent results. This phenomenon, may be due to different behavior of polymorphism in various races. However, environmental and nutritional factors are also involved in the effect of this polymorphism.

Folate, a form of vitamin B9, is found chiefly in plant diets. The human cell requires folate as a cofactor for numerous reactions, such as DNA synthesis, DNA methylation, and DNA repair (Ericson et al., 2009). Then diets deficient in fruits and vegetables are generally low in folate, antioxidants, and a lot of other micronutrients which leads to significant amounts of DNA damage and higher cancer rates (Blount et al., 1997). Low consumption of folate and related B vitamins plays as one of the few risk factors associated with breast cancer (Chen et al., 2005). Some studies revealed that folate intake to be inversely associated with the risk of breast cancer (Campbell et al., 2002; Ericson et al., 2009). Wang et al., (2014) and Weiwei et al., (2014) found a significant association of MTHFR-C677T transition and folate intake with risk of breast cancer, and they reported a significant interaction between folate intake and MTHFR-C677T transition (Wang et al., 2014; Weiwei et al., 2014). Also, Ericson et al., (2009) suggested a significant association of high concentration of folate with the risk of postmenopausal breast cancer risk in carriers of the MTHFR-677T allele. Generally, the role of folate to the prevention of tumor development before preneoplastic lesions have been proven, but it conversely increases tumorigenesis once lesions. However, increase the folate intake in women with already-sufficient levels of folate may be harmful (Ma et al., 2009). Shrubsole et al., 2004 evaluated C677T and A1298C variations and their impacts on the folate intake and association of breast cancer susceptibility. They reported an inverse correlation of breast cancer risk with the intake of folate for all genotypes, especially among subjects with the 677TT genotype. They suggested that MTHFR-C677T variation might modify the correlation of dietary folate intake with breast cancer risk. But, there was no modifying effect of A1298C genotypes on the correlation of folate intake with the risk of breast cancer (Shrubsole et al., 2004). 

Consequently, the MTHFR enzyme has a balancing role in the pool of methyl groups for DNA methylation, protein methylation, and DNA synthesis (Ericson et al., 2009). Thus, MTHFR activity may affect both gene expression (by DNA methylation), and genome integrity (by DNA synthesis and repair) (Le Marchand et al., 2004). The potential impact of MTHFR enzyme activity on DNA methylation, synthesis, and repair make it a possible candidate in cancer-predisposing (Campbell et al., 2002). The SNP’s effects on the molecular aspects of a gene were dependent on its gene location (Karimian et al.,(2020); Mobasseri et al., 2019). Evaluation of these effects by in vitro and in vivo methods is very time and cost consuming (Zamani-Badi et al., 2018; Bafrani et al., 2019). Assessment of the mentioned impacts and other molecular assessments by in silico tools may be too beneficial (Noureddini et al., 2018; Tameh et al., 2018). A study with bioinformatics analysis revealed that both C677T and A1298C genetic variations made significant changes in the secondary structure of MTHFR-mRNA. Moreover, a structural examination of the A1298C polymorphism showed a significant impact on the function of MTHFR (Salimi et al., 2017).

In conclusion, based on our findings, the MTHFR-A1298C and MTHFR-C677T variations could be genetic risk factors in our studied population. But, these polymorphisms could not be considered as genetic risk factors for tumor size and lymph node metastasis in our studied population. However, there are some limitations in our study, for example, a small sample size in the case-control study especially in the stratified analysis is the main limitation. Also, lack of evaluation of folate and homocysteine level is another limitation of our project. On the other hand, lack of attention to gene-environment and environment-environment interactions are other limitations of our study. However, further studies with a larger sample size regarding the aforementioned interactions could be helpful to obtain more accurate data.
